# Quality of care for Black and Latina women living with HIV in the U.S.: a qualitative study

**DOI:** 10.1186/s12939-020-01230-3

**Published:** 2020-07-06

**Authors:** Whitney S. Rice, Faith E. Fletcher, Busola Akingbade, Mary Kan, Samantha Whitfield, Shericia Ross, C. Ann Gakumo, Igho Ofotokun, Deborah J. Konkle-Parker, Mardge H. Cohen, Gina M. Wingood, Brian W. Pence, Adaora A. Adimora, Tonya N. Taylor, Tracey E. Wilson, Sheri D. Weiser, Mirjam-Colette Kempf, Bulent Turan, Janet M. Turan

**Affiliations:** 1 Department of Behavioral, Social and Health Education Sciences, Emory University, 1518 Clifton Road NE, Atlanta, GA 30322 USA; 2 Department of Health Behavior, University of Alabama at Birmingham, Birmingham, AL USA; 3 Hubert Department of Global Health, Emory University, Atlanta, GA USA; 4 Department of Psychology, Emory University, Atlanta, GA USA; 5 Department of Health Care Organization and Policy, University of Alabama at Birmingham, Birmingham, AL USA; 6 Department of Nursing, University of Massachusetts Boston, Boston, MA USA; 7 Department of Medicine, Emory University, Atlanta, GA USA; 8 Department of Medicine and School of Nursing, University of Mississippi Medical Center, Jackson, MS USA; 9 Department of Medicine, Stroger Hospital of Cook County, Chicago, IL USA; 10 Department of Sociomedical Sciences, Lerner Center for Public Health Promotion, Columbia University Mailman School of Public Health, New York, NY USA; 11 School of Medicine and UNC Gillings School of Global Public Health, University of North Carolina at Chapel Hill, Chapel Hill, NC USA; 12 Department of Community Health Sciences, State University of New York, Downstate Health Sciences University, Brooklyn, NY USA; 13 Division of HIV, Infectious Disease and Global Medicine, Department of Medicine, University of California, San Francisco, San Francisco, CA USA; 14 School of Nursing, University of Alabama at Birmingham, Birmingham, AL USA; 15 Division of Infectious Diseases, University of Alabama at Birmingham, Birmingham, AL USA; 16 Department of Epidemiology, University of Alabama at Birmingham, Birmingham, AL USA; 17 Department of Psychology, University of Alabama at Birmingham, Birmingham, AL USA

**Keywords:** Quality of health care, Patient satisfaction, Women living with HIV, Engagement in care, HIV/AIDS, Qualitative, African American, Black, Hispanic

## Abstract

**Background:**

Ending the HIV epidemic requires that women living with HIV (WLWH) have access to structurally competent HIV-related and other health care. WLWH may not regularly engage in care due to inadequate quality; however, women’s perspectives on the quality of care they receive are understudied.

**Methods:**

We conducted 12 focus groups and three in-depth interviews with Black (90%) and Latina (11%) WLWH enrolled in the Women’s Interagency HIV Study in Atlanta, GA, Birmingham, AL, Brooklyn, NY, Chapel Hill, NC, Chicago, IL, and Jackson, MS from November 2017 to May 2018 (*n* = 92). We used a semi-structured format to facilitate discussions about satisfaction and dissatisfaction with health care engagement experiences, and suggestions for improvement, which were audio-recorded, transcribed, and coded using thematic analysis.

**Results:**

Themes emerged related to women’s health care satisfaction or dissatisfaction at the provider, clinic, and systems levels and across Institute of Medicine-defined quality of care domains (effectiveness, efficiency, equity, patient-centeredness, safety and timeliness). Women’s degree of care satisfaction was driven by: 1) knowledge-based care resulting in desired outcomes (effectiveness); 2) coordination, continuity and necessity of care (efficiency); 3) perceived disparities in care (equity); 4) care delivery characterized by compassion, nonjudgment, accommodation, and autonomous decision-making (patient-centeredness); 5) attention to avoiding side effects and over-medicalization (safety); and 6) limited wait time (timeliness).

**Conclusions:**

Quality of care represents a key changeable lever affecting engage in care among WLWH. The communities most proximally affected by HIV should be key stakeholders in HIV-related quality assurance. Findings highlight aspects of the health care experience valued by WLWH, and potential participatory, patient-driven avenues for improvement.

## Background

Women represented 19% of the 37,881 new HIV diagnoses in the United States in 2018 [1], and 23% of the estimated 1.1 million people living with HIV in the U.S. [2]. Evidence suggests that for every 100 U.S. women living with HIV, approximately 66 received some HIV medical care, 51 remained in continuous HIV care, and 53 were virally suppressed – all critical indicators of well-being and population-based HIV treatment and prevention [3]. Black and Latina women in the U.S. in particular are less likely to use antiretroviral therapy (ART) [4], less likely to be virally suppressed [5], and more likely to die of causes related to HIV [6], as compared to white women. These inequities are in part attributed to compound systemic barriers to adequate HIV and non-HIV care faced by racial and ethnic minority women. Racial and ethnic minority women living with HIV (WLWH), are socially positioned at the juncture of multiple marginalized identities (i.e., as people who identify as Black and/or Latina, as women, and as people living with HIV, in addition to socioeconomic status, immigration status and other social statuses), and encounter both multilayered intersectional stigma and systemic oppression with regard to their social position (i.e., racism, sexism, classism, etc.) – including within health care systems [7]. Ending the HIV epidemic requires that Black and Latina WLWH have access to structurally competent health care, or care that is attentive to the structural factors that produce health inequities (i.e., factors that “codify differential access to social, political and economic opportunities”) [8].

Existing literature documents inadequate quality of care (care that is not adequately informed by professional evidence, and that does not adequately produce needed and desired health outcomes [9]), as one of the reasons why minority WLWH may not engage in HIV care or other health care services [10, 11]. Quality of care is a multi-dimensional concept that has evolved over time. Early characterization of quality of care by Donabedian in 1978 focused on a few fundamental characteristics: structure (e.g., staffing, physical space, equipment, etc.), process (i.e., evidence-based methods of delivering health care – from appropriate medication administration, to infection prevention to follow-up care provision), and outcomes (i.e., patient health status and care experience – including quality of life, length of hospital stay, readmissions and mortality) [12]. In 1984, Maxwell expanded these concepts to include six additional dimensions: access to services, relevance (to community need), effectiveness (for individual patients), equity (or fairness), social acceptability, and efficiency and economy [13]. In 1998, the U.S. National Academy of Medicine, formerly the Institute of Medicine, characterized quality care in complementary, simplified and well-defined terms, as the degree to which health care is *effective* (provided as informed by science and to those who would benefit), *efficient* (avoids waste), *equitable* (unvarying in quality by patient characteristics), *patient-centered* (respectfully and responsively guided by patient preferences, needs and values), *safe* (avoids patient injury), and *timely* (limits wait and delay) [14]. We refer to the National Academy of Medicine framework throughout this paper for consistency, since it encompasses earlier frameworks, and because it has informed standards and recommendations by leading health agencies such as the World Health Organization and the U.S. Agency for Healthcare Research and Quality [15, 16].

Women living with HIV in the U.S., compared to their male counterparts, historically receive lower quality of care for managing HIV and other chronic conditions [17]. For example, women are historically less likely to receive important HIV-related care services such as drug resistance testing, pneumonia prophylaxis, CD4 and viral load tests, and hepatitis C testing as compared to men [18–20]. Policy and programmatic gaps such as limited policy assurance of equal access to HIV-related and other health services, lack of policy protections against delivery of ineffective services, and inequities in comprehensive HIV knowledge [17, 21], hinder the provision of more equitable quality of care for racial and ethnic minority women globally. Considering these multilevel inequities in care quality and other factors, an emerging body of scientific literature calls for intersectional women-centered health care models of that meaningfully involve WLWH in health services design, delivery, and evaluation [22–24]. This evidence suggests that the alignment of health care delivery with the needs and desires of the WLWH, who use health services and for whom services are intended, may better address persistent racial and gendered inequities in health vulnerability, care quality, and related outcomes, relative to less participatory care processes. Women-centered models of care should then ideally follow a health care quality priority setting and quality improvement process that is responsive to the perspectives and knowledge of WLWH, and that engages WLWH [25].

The current study aims to contribute to this emerging evidence base by investigating U.S. women’s perspectives on the quality of HIV and other health care services. Few published research studies explicitly evaluate quality of care from the perspective of WLWH [26]. Existing quantitative studies that explicitly examined quality of care in the U.S. among WLWH used clinical quality indicators extracted from the medical record [18, 20]. Others quantitatively assessed women’s satisfaction with medical care generally, within specific specialties (e.g., obstetrics and gynecology) and with specific domains of care quality (e.g., care accessibility) [27–29]. Domestic qualitative studies in this area largely do not evaluate quality of care explicitly nor exclusively, and present findings related to few dimensions of care quality [10, 11, 30]. Here, we contribute a qualitative exploration of perceptions and lived experiences across a comprehensive range of care quality domains (i.e., effectiveness, efficiency, equity, patient-centeredness, safety, and timeliness*)* among Black and Latina WLWH in six U.S. cities to inform patient-centered and participatory quality improvement efforts.

## Methods

### Study design

The present analysis is part of an exploratory mixed-methods sub-study to the Women’s Interagency HIV Study (WIHS) [31] to examine the effects of stigma and discrimination in health care settings (critical barriers to quality health care [32]) on patient-provider interactions, engagement in care, treatment adherence, and viral load among women living with HIV. Considering the disproportionate HIV and HIV stigma burden faced by Black and Latina women as compared to other racial and ethnic groups in the U.S., the initial qualitative phase of the study was intentionally designed to engage the perspectives of Black and Latina women living with HIV only. The qualitative study phase aimed to explore stigma and discrimination (due to HIV, race/ethnicity, and other intersectional identities), concepts interrelated with quality of health care, as they impact key health care interactions for Black and Latina women living with HIV via 12 focus groups (of 5–11 participants per group) and 3 individual in-depth interviews conducted in English or Spanish. Focus groups served as the primary method of qualitative data collection, in order to foster intergroup discussion regarding stigma, discrimination, and health care interactions among Black and Latina women living with HIV. Interviews supplemented focus groups in order to engage women in the study who preferred to participate using Spanish at locations where we were not able recruit enough Spanish-speaking women to hold a focus group. Findings from the qualitative phase also served to inform the refinement of measures added to the national WIHS cohort questionnaires to quantitatively assess associations of stigma and discrimination in health care settings with adherence, engagement in care, and viral load suppression.

### Study sampling and recruitment

Women were recruited from five WIHS sites located in (1) Birmingham, AL (University of Alabama at Birmingham) and Jackson, MS (University of Mississippi Medical Center), which form the combined UAB/UMMC WIHS site; (2) Atlanta, GA (Emory University); (3) Brooklyn, NY (State University of New York Brooklyn); (4) Chapel Hill, NC (University of North Carolina at Chapel Hill [UNC]); and (5) Chicago, IL (Stroger Hospital of Cook County). Trained WIHS staff verbally informed potential participants of the focus group opportunity. Women who expressed interest in the study were in turn invited to participate at pre-scheduled focus group and interview times at participating sites. The study recruitment and consent processes described the study interest in understanding patient-provider interactions from the perspectives of women living with HIV for tailored policy and intervention development to improve HIV care and to help women lead long and healthy lives. We enrolled women into focus groups and in-depth interviews between November 2017 and May 2018. Participants were compensated $50 for their time and engagement in the study.

### Data collection

Experienced, qualitatively trained, female interviewers and focus group moderators conducted all focus groups in English and all individual interviews in Spanish. Discussion was guided by the same semi-structured focus group and interview guide, translated to Spanish for the interviews (see Additional Files 1 and 2). Interview questions included, “What kinds of experiences do people living with HIV have in health facilities?” “What kind of things do women living with HIV worry about when they visit a health care facility?” and “What could health care providers or other support services do to make it better for you/or other women?” At the beginning of the focus groups and interviews, we provided the following definition of the term “health care provider” for context: *By a health care provider, I mean a doctor, nurse, physician’s assistant, social worker, pharmacist, dentist, or other person that provides you with services at a doctor’s office, hospital, clinic, or pharmacy.* Moderators and interviewers used discussion probes when necessary to encourage participants to elaborate on their responses. Focus groups lasted approximately 90 min each and the interviews lasted a half hour, and were audio recorded. Audio recordings were transcribed verbatim by an experienced transcription company. Spanish language transcripts were translated into English prior to analysis.

### Data analysis

We analyzed transcripts using a two-stage inductive thematic analysis process [33]. In stage 1, a team of five researchers (WSR, FEF, SR, CAG, JMT) developed an initial framework of broad codes and sub-codes in which to organize the data based on patterns that emerged during review of the initial transcripts, reflection on the primary study aims, and considering literature regarding stigma and discrimination in health care settings. Three members of the study team (FEF, SR, CAG) coded the transcripts using NVivo 12 software, initially double-coded transcripts, compared double-coded transcripts, and then discussed and resolved discrepancies with the larger analysis team to reduce variety in approaches to analysis and to maximize reliability.

In the second stage of analysis, the analysis sub-team revised the initial codebook on this basis and coded the remaining transcripts. Two research team members (WSR, MK) conducted a secondary analysis of the coded data to explore themes related to quality of care. The initial codebook included care quality relevant codes that emerged during analysis such as: “recommendations to improve health care experience”, “advice for other PLWH”, “continuity of care”, and “compassionate…, respectful, non-judgmental” care. Through additional independent double-coding, comparison, and discussion, new emergent codes (“Quality of Care”, “Satisfaction”, “Dissatisfaction”, “Patient-Centeredness”, “Equity”, etc.) were added and applied to further categorize and expand upon the originally broad coded data with respect to care quality. Preliminary results from this analysis specific to quality of care were presented iteratively to the original analysis team, to other collaborators and to external experts for peer debriefing prior to finalization for the present manuscript. During the writing process and after coding was complete, we organized the resulting themes by the National Academy of Medicine quality of care domains (i.e., effectiveness, efficiency, equity, patient-centeredness, safety, and timeliness).

## Results

Participants were predominantly Black (90%), non-Hispanic or -Latina (89%), over the age of 50 (57%), and had lived with HIV for 10 or more years (53%), as seen in Table 1. Themes from the study discussions that were identified during data analysis are described below and illustrated by the quotes in Table 2, primarily organized by quality of care domains. Notably, in some cases, participants discussed quality of care themes simultaneously. Thus, multiple quality of care domain codes were applied to some quotes.

**Table 1:** Characteristics of study focus group and interview participants

	92 (%)
**Race**	
Black	83 (90%)
Non-black*	9 (10%)
**Ethnicity**	
Hispanic or Latina	10 (11%)
Not Hispanic or Latina	82 (89%)
**Age Group**	
< 50 years of age	40 (43%)
50+ years of age	52 (57%)
**Time since HIV Diagnosis**^†^	
< 10 years	26 (28%)
10+ years	60 (65%)
Unknown/Not Reported	6 (7%)
**Educational Level**	
< High School/GED	27 (29%)
High School/GED	30 (33%)
Some College/Associate	23 (25%)
College and above	9 (10%)
Unknown/Not Reported	3 (3%)
**Monthly Income Range**	
$1000 or less	49 (53%)
$1001–2000	25 (27%)
$2001–3000	8 (9%)
$3001+	8 (9%)
Unknown/Not Reported	2 (2%)
**WIHS Location**	
Atlanta, GA	16 (17%)
Birmingham, AL	19 (21%)
Brooklyn, NY	14 (15%)
Chapel Hill, NC^a^	14 (15%)
Chicago, IL	18 (20%)
Jackson, MS	11 (12%)

* Includes women who responded ‘brown,’ ‘biracial,’ or ‘more than one race’

† Based on the time of interview.

^a^ The UNC site enrolled 11 women in focus groups as well as 3 Hispanic/Latina women for in-depth interviews

**Table 2:** Illustrative quotes by quality of care domain, sub-theme and satisfaction category

Care Quality Domain	Sub-Theme	Experience ^**a**^	Quote
Effectiveness	Knowledge-Based Care	General	In response to the question, “What are you looking for [in a health care provider]?”: *Somebody that is knowledgeable, that knows what she’s talkin’ about in terms of medications and treatments and stuff like that. Somebody that knows.*
		Satisfaction	*He’s very educational when it comes to the different medications and different programs out there that might help me. Usually, if I have a problem that comes up, the medication he gives me knock it right out … I know my body, and it doesn’t feel right, …I will complain to him. He will say, “Okay, we gonna test you for this,” and then come to find out I might have a nerve problem or somethin’ like that.*
	Desired Care Outcomes	Satisfaction	*My pressure was so high ... I went to the emergency room. Them folks kept me in there overnight… They kept running stuff, and then they finally gave me some medicine, and my pressure went down, but they take their time. They ain’t fit’n to rush and just come in there and go, “Well, we ain’t find nothin’ in your labs. Well, everything good,” and send you on about your way. They’re gonna make sure.*
		Dissatisfaction	*I have issues with them because … I get headaches real bad, and they told me everything looked good, but I still have the headaches so bad. With these doctors from other hospitals, not here, they started giving me this MIRs and different head scans and stuff, and keep telling everything looked good, but I constantly have these.*
Efficiency	Continuity of Care	Dissatisfaction	*As far as me being comfortable with one provider, and then the idea of them switching providers and not giving you a notice, that’s awful hard when somebody walk in the room. You sittin’ there, you’ve been there maybe a hour, 45 min waiting there. Then the stranger is [there, and] you got to start back over with somebody, opening up your heart.*
	Care Coordination	Satisfaction	*He’s been so respectful and so helpful for me. Just like last time I went the hospital. Pressure was high, and next thing I know the doctor called, my doctor from here called and my social worker called, and even with my high blood pressure… I’ve got a blood pressure partner that calls to check, how is my pressure, have I took my medicine today, or something like that.*
		Dissatisfaction	*I had the same experience with the pharmacy. The HIV-positive medication. Always tellin’ me I don’t have refills. I’m like, "How can that be?" Then I call my doctor and then the secretary tell me, "No. The pharmacy has to call me." Then I call the pharmacy and tell the pharmacy and the pharmacy tells me, "Well, tell them to call us." I’m like, "Listen. Y’all gotta get this shit together. Who am I supposed to call to get my medicine?" It should not be that difficult. If I need a refill, pharmacy should be able to call the doctor’s office or send them a email, whatever it is that you could do. Everything is electronically now. It shouldn’t be a problem. Press one button and ask for the refills. It’s not like I’m new. I’m still three days with no medicine.*
	Necessity of Care	Dissatisfaction	*One time, I had gone to the emergency room … because I thought I was having a heart attack, which turned out to be an anxiety attack, which was all figured out three days later, after me being in the hospital three days.*
Equity	Differential Treatment	Satisfaction	*The doctors, they didn’t treat me no different. To this day, they’re still the same.*
		Dissatisfaction	*I had one woman who is the first one who is really not friendly at all. No kinda bedside manner. Nothin’ like that. She was kinda halfway scared to touch ya… [HIV] is bad enough… I don’t wanna talk to the doctor and feel like that too.*
		Dissatisfaction	*Well, I had gotten sick with pneumonia. I went to [hospital]. Biggest mistake I could’ve ever made. When they found that I had HIV, they did not wanna treat me. They would not even examine me.*
	Differential Resource Allocation	Dissatisfaction	*Why they gettin’ all these funding, but every time I ask for somethin’, there’s—they cut the budget.... There’s no funding for that… You go over in the adolescent program, they treatin’ them like kings and queens. I mean, I get that they’re young, but—we dealin’ with the same issues here.*
Patient-Centeredness	Shared Decision-Making	Satisfaction	*She’s great. She’s a great listener. Whatever direction you wanna go, she’s there. She’s supporting whatever decisions that you make, and whatever help, whatever’s going with you, and if it’s not within [her clinic], and if it’s something else, she’ll do it. If you need a referral or something, she’ll do it. She’s really wholesome.*
		Dissatisfaction	*I’m at the behavior clinic here, I got this [provider]—and think he knows everything too…. He got his opinion about somethin’, and I got my opinion. He talkin’ my way is a fact, I told him, “No, it’s your fact.” I really don’t wanna hear what he was talkin’ about…*
	Compassionate Care	General	*You do wanna be with a provider that you feel like, “All right, he’s looking out for my best interest,” not, “Oh, he’s lookin’ out to do the surgery so he can make this amount of money off of me.” See, that’s not the type of feeling you want from somebody and that’s the only thing. You just want people to at least have that empathy or at least be able to sympathize with you sometimes and understand where you’re comin’ from.*
		Satisfaction	*Yeah, I had some good experience, too. The psychiatrist I found—see, that doctor, the psychiatrist, she was so humble. I was goin’ through somethin’ deep. She came to my house. I was shocked. I said, “Oh, y’all do house calls?” Then she came to visit me in the hospital. I had no support.*
	Nonjudgmental Care	Satisfaction	When asked: “what are some things that are important in a relationship with your provider?” *Well, definitely that she listens. And she’s open to whatever concerns I may have, even if it’s something that I have reoccurring, that she’s bashed me over the head about doing or not doing. And she’ll still come to me with open arms and just say, "Well okay, you have to keep in mind that this is what’s gonna happen, but you still come back, you still call me, and don’t worry about anything like that."*
		Dissatisfaction	*When I tell you that I haven’t taken my meds for six months, or I don’t even know the last time I’ve taken my meds, and you go, "Well, what’s wrong with you? Why would you do that?" …. I could tell when you’re coming down on me because of the fact that you actually genuinely care, or you feel like you just feel the need to just come down on me because you think I’m stupid and you think I’m crazy. You’re not even trying to figure out why it is that I just have these moments when I just stop doing this. It’s a depression thing for me…. It’s a moment in time. Sometimes the moment could last a last couple of days, couple of weeks, couple of months, but it’s a moment in time. My provider, if you know me, you know that I have had a timeframe…. That’s the connection for me. That’s what matters the most, if you can be understanding, nonjudgmental, and show me that you actually care about what’s going on with me.*
	Care Accommodation	Dissatisfaction	*I tell them give me the appointment for early, because my health aide is with me from 9:00 to 1:00. They wanna give me the appointments at 3:30, 4:00. Listen, I like to do everything early because after a while, my body starts to shut down.... No, I don’t wanna come to the hospital at that time.*
		Satisfaction	*I think the best thing, as far as with HIV care and just the accommodations are generally someone of my size can’t find a scale. I have to walk further places. When they know that I’m coming in, they do go above and beyond. When I go, it’s very busy. I know that they’re coming out of their comfort zone, in order to accommodate me. I really appreciate that. It makes the visit go better.*
Safety	Side Effect Prevention	Satisfaction	*Like I said, I’m satisfied with her, and she’s a good doctor. She recommended that after seven years, and I changed my medication due to a—that it would take effect on my kidneys after so long. She did switch my medication with my permission.*
		Satisfaction	*…they just switched my medicine cuz they said I’ve been on the other one too long, and I think it had just something to do with my bones. It made them brittle cuz they said I’ve got osteoporosis now.*
		Dissatisfaction	*Even though the medicine is keeping us alive, there’s still drawbacks to it. It affects our liver, it affects our kidneys. It affects our hearts. Just thinking about that … in and of itself has been a struggle...*
	Overmedication	Dissatisfaction	*You wanna apply medication on top of medication, on top of medication. My pastor always told me, just cuz the doctor provide it to you don’t mean you have to take it.*
		Dissatisfaction	*When I get ready to go pick up my meds, they done put some more. What is this for? I’ve already been on medicine for so long. Then you give me some more medicine. I’m like, ‘I’m not fit’n to take this cuz you prescribed it for me, and you gave it to me, but you didn’t inform me of it.*
Timeliness	Care Wait Time	Satisfaction	*…what I like about them, they don’t give everybody a 9:00 appointment. If I get there at 9:00, 9:05, they’re calling me... If your appointment is at …9:30, okay, at 9:35, I’m out that office at 9:35, and you’re walking in at 9:35. You’ll go walking in five minutes after the time you get there when you’re going to see a doctor because everybody don’t have a 9:00 appointment like they do here.*
		Dissatisfaction	*The only problem I really have is when I go, and I got a 9:00 appointment, and then they don’t see me ‘til 10:30. Don’t give me no 9:00 appointment, cuz I’m expectin’ to see doctor at 9:00... I have other things to do, so [I] set aside this time to come see you.*
		General	*…when I come there, I comin’ there get my medicine, you check me, let me know if anyhthing wrong me, and get me the hell outta there cuz I don’t like bein’ there no way… I don’t like bein’ where I know a lotta people. It just somethin’ about seein’ people where I go that does somethin’ to me*… *You know they talk.*

a Refers to patient care experience, categorized as greater satisfaction, dissatisfaction, or general (neither satisfaction or dissatisfaction)

### Effectiveness

The women who participated in our study mentioned multiple criteria for their health care satisfaction related to whether care was knowledge-based and resulted in desired care outcomes. Specifically, women expressed considerations around provider qualifications for provision of HIV and other health care services, the identification and use of best treatment practices, and the achievement of HIV-related and HIV treatment outcomes, as seen in Table 2. Women shared appreciation for circumstances where doctors communicated and sought resolutions, even when they lacked expertise around a course of action, as one participant imparted, “I have COPD [Chronic Obstructive Pulmonary Disease] and the HIV. COPD, certain medicine [interacts with HIV medicine]—if [my provider] don’t know—what I like about her, she will call down to pharmacies, and she’ll see which one [interacts], cuz I done had a doctor where they didn’t care. They just say, "Okay, I’m a give you this right here.”

Women generally reflected positively upon health care experiences where they walked away with a treatment plan to improve their health condition or alleviate health concerns. On the other hand, participants expressed that prescribed treatment plans that did not produce better health outcomes contributed negatively to patient satisfaction, patient-provider relationships and potentially, mental well-being. In a statement mirroring that of other participants across focus groups and study sites, another participant shared: “They’ll help you with anything but pain. That’s the biggest problem…. I have spoken freely to my health care provider. It didn’t go well. I told him, ‘You givin’ me Tramadol. Tramadol is not workin’ for me.’ … I want you to know three days later, the nurse emailed me and said, ‘He gonna put you on Tramadol.’ It didn’t work! I told him when I was in his face, it didn’t work. I made this statement to like three doctors… [and] ‘I understand why people commit suicide because of pain... They just want the pain to be over.’” These experiences with inadequate pain management were reported by women regardless of history of substance use.

### Efficiency

Women in this study discussed their satisfaction and dissatisfaction with few aspects of the coordination, continuity and necessity of care received – in particular the flow and organization of the health care. The most prevailing concern was continuity of care, or the degree to which women were able to consistently engage with the same health care providers. Some participants in both the interviews and focus group discussions reported feeling satisfied with their relationships with their health care providers due to the long-term nature of their relationships in which strong rapport and a level of comfort and trust were established. When a change in provider occurred due to planned or unexpected leave or relocation, or clinic staffing changes, many women described that they did not appreciate having to establish a new relationship. As a whole, most women reported not being aware that their providers were leaving the health facilities they attended or could otherwise no longer treat them. A minority of women shared that their previous providers or the health facilities made them aware in advance of an anticipated interruption in service as previously provided (See Table 2).

Women from each of the study sites shared reflections on the level of care coordination by provider across different care facilities and specialties. One participant noted: “I get along with mines good. They’re good to me. They’ll call, check on me on a regular; nurses, health care social worker.” Overall, most women highly valued providers that proactively communicated with the other providers to advocate for their specific health care needs. For example, one participant expressed appreciation for her pharmacist for proactively reaching out to her physician to renew expiring prescriptions on multiple occasions. Participants noted that when their care was not well-coordinated, they bore the consequences, including delayed receipt of medication and care and excessive or unnecessary care.

### Equity

Participants also reflected upon differential treatment by health facilities, providers, and health systems due to their HIV status. Few participants across sites stated that they were unfamiliar with stigma and discrimination (as seen in Table 2). The majority of participants shared experiences of provider discrimination, such as the excessive use of gloves, masks and other protective gear, avoidance of contact altogether, patient isolation, and in a few instances - denial of care. Participants viewed these practices as unnecessary and questioned whether similar practices were employed by providers with HIV-negative patients. Women who were denied care added that these experiences can affect women emotionally, and ultimately their health care seeking behaviors and practices (i.e., whether, when and where to engage in health care services), as a participant added: “One time, I went to the dentist.... They ask about your status and all that. I did put on there that I was HIV-positive. When they got back in the room, they said that they couldn’t work on me.... They recommended that I go to the dental school…” When asked how this experience made her feel, the same participant responded, “Very, very bad. I quit takin’ my daughter over there, too.”

A few participants also shared the perception that fewer programmatic and health care system resources are being devoted to them as older adult and Black WLWH compared to overemphasis on the needs of younger, male, largely white, and opioid using populations. Correspondingly, a participant shared her perspective regarding systemic ageism: “when we was 18, it was a whole different ballgame. Now, a 18-year-old that would come in the clinic, it’s like they’ll give them more attention because they tryin’ to stop this epidemic. See? Back when we was 18, heck, they didn’t care. We just passin’. Now, they tryin’ to do this intervention and tryin’ to keep less people from gettin’ it. To me, they give a younger person way more attention.” Women expressed frustration with resource allocation and the notion that women and older adults are in less need of health care services.

### Patient-centeredness

Relative to the other care quality domains, participants most frequently shared perspectives on the degree to which the health care that they receive is responsive, respectful, and guided by their needs and preferences. Most of the women in the study spoke highly about their health care encounters and communicated that their needs are generally prioritized. The women in our study also valued unrushed and attentive care. Health care providers, who showed concern for their patient’s overall health and well-being were held in high regard by study participants. As one participant described:, “when I walk through the door, ‘Hi. How are you feeling?’ ‘I’m all right.’ ‘How’s the family? How’s your grandkids? I know they got big. Let me see some pictures.’ We’ll see pictures. We’ll talk about this, how I’m doing. Before we get to my health, she wanna know how I’m doing in my relationship. How I’m feeling mentally.”

Participants also viewed provider compassion as one key characteristic of patient-centeredness. A participant elaborated on how her health is positively affected by these compassionate and caring provider relationship dynamics: “When you walk in, they throw lovin’ arms around you, and treat you so good. I used to always go to the doctor and have high blood pressure. Now when I go, I feel so comfortable with ‘em because they’re so kind, and everything. My blood pressure’s always great.”

Relatedly, patients expressed satisfaction when providers were understanding and nonjudgmental of their behavior, which directly affects women’s comfort with and willingness to speak openly with providers: “My doctor and I have a good relationship because she’s nonjudgmental …. Even if it’s the lowest thing I ever done in my life, I feel I can share with her. I remember at my first doctor, I would keep it to the bare minimum because I didn’t want her to judge me. Then the doctor I’m with now, I could go in there and just practically say anything. She say, ‘Okay, [participant name].’ Then we’ll discuss it. Not one time she’ll frown or shun or frown, whatever situation it is. We discuss things. I like that about my provider.” Women also discussed instances where they perceived that providers lacked compassion, were judgmental, or were not genuinely concerned about their well-being, which women also saw as consequential to their patient-provider relationships.

Participants additionally expressed satisfaction when providers respected and prioritized their preferences and needs to inform health decisions. The women in our study valued providers that treated them “as their equal”. Dissatisfaction was expressed when women in the study felt their opinions were not valued, as indicated by disregard of their preferences and decisions regarding their own health care. Women expressed the perception that some providers see themselves as the sole authority in decision-making and they wanted to have more of a say in their health care: “I think they assume things. I went to an orthopedic doctor maybe three months ago. He did an x-ray. He told me what I already knew. I had arthritis in my joints. He didn’t really offer anything except an injection. Didn’t ask me if I wanted the injections, how I felt about ‘em. He came in with a needle, and like, [I said] ‘What are you doin’?’ He goes, “Well, this is dah-dah-dah-dah-dah, and we’re gonna put it in you.” I said, ‘No, we’re not.’ Again, don’t assume that you’re gonna be doin’ anything to me.”

Furthermore, participants appreciated when care was accessible and tailored specifically to their health needs. More specifically, women expressed satisfaction with clinical environments that provide appropriate space, equipment and capacity for patient access and use regardless of their body size and mobility (see Table 2). Women discussed that when care did not accommodate special needs of women with co-morbidities, there were potential consequences such as difficulty adequately engaging in care: “I used to be able to get around really well. Now, not so much. That has become a problem for me. Some of the providers, they don’t take into account that I have difficulties with that. If I’m gonna be at [facility] for an appointment, I’m either need to be dropped off, or I’m gonna need to make all my appointments the same day, cuz I can’t be going all the way from the parking lot, in the cold, down, cross the street, down, up to [facility]…. Then, even if I could get a shuttle, I’d still have to sit out in the cold, with my old tired bones, waiting for it. No.”

### Safety

Discussion of provider attention to safety (e.g., avoiding infection, medical error, side effects and over-medicalization) and its’ impact on satisfaction with their health care experience emerged in fewer instances. When asked, “What kinds of things do women living with HIV worry about when they visit a health care facility?” Responses included “infections”, “cleanliness” and “bacteria”. Participants across groups mentioned concerns that the health facilities that they go to for care are not sterile or appropriately clean, particularly considering that WLWH are susceptible to infection. Most participants were satisfied with health care interactions that prioritized their immediate and long-term health including preventing long-term harm from medication use. For example, participants appreciated proactive efforts demonstrated by their providers such as changing prescription regimen in order to avoid potentially harmful side effects, as noted in Table 2.

On the other hand, participants complained of not being adequately informed about changes to their regular medication regimen. A couple of participants expressed the concern that new medications were being tested on them without their permission, contributing to medical mistrust skepticism surrounding treatment recommendations. Finally, a few participants described experiences with more severe medical errors, including instances of severe reaction to a newly prescribed medication and being prescribed another patient’s medication. As a participant shared: “She gave me some medicine. There’s another girl that had the same name as mine. She gave me her medicine… wasn’t no HIV meds. I got sick. I passed out right there in the doctor’s office. My doctor fired her on the spot. Right there. Told get her, excuse my language, but so and so, get out.”

### Timeliness

Lastly, participants consistently commented on how well their time was managed in the health care setting. Women primarily discussed their satisfaction with the wait times during clinic visits, and with the length of time in between their clinic appointments (see first timeliness quote of Table 2). Most women appreciated being seen promptly after arriving and checking-in or at a digital patient check-in kiosk. Women who expressed dissatisfaction with long wait times at their clinics shared concerns about lack of confidentiality and privacy in the waiting rooms, given anticipated stigma from others, as seen in the last quote of Table 2. However, some of the women added that breaches in confidentiality were more common concerns when they were newly diagnosed but that these concerns ultimately dissipated over time.

Women preferred to maximize their time by balancing other life demands to the extent possible. For some participants, long wait times were a deterrent, especially considering the total time to travel to a clinic and the appointment itself. As expressed by a study participant, “It’s just that I just don’t have time. I live an hour and a half away ... Time is of the essence for me. I need to go.” For these reasons, women appreciated clinics that clearly communicated upfront the expectation that a provider would be running late or that offered to cancel and reschedule appointments when clinic flow was slower than anticipated. Some women also appreciated the opportunity to have less frequent clinic visits to reduce time and travel burden.

Study participants also spoke highly of clinics that allowed some flexibility in the scheduling and rescheduling appointments in a short timeframe when needed. Another participant illustrated: “I missed mine yesterday, and I got an appointment that’s [the following month] … that’s pretty expedient… the [Office Worker] called me yesterday, said that he’ll work me in…, ‘[month] is the first day I have open’… so I took it… I was satisfied ...” On the other hand, women discussed dissatisfaction with care delays due to a lack of available appointments. Participants described having to wait three to four months to be seen by specialists when they perceived specialty care as urgent and essential to managing chronic conditions such as diabetes. Participants expressed concerns that delays in specialty care could be harmful to their health.

### Patient-driven quality improvement and self-advocacy

Participants voiced recurring suggestions for future care quality improvement at multiple socio-ecological levels. At the provider level, women expressed the need for provider education (including continuing sensitivity and patient-provider relationship training), provider-patient communication, respect for patients, patient bedside manner, and empathy for patients to improve quality of care. At the clinic and systems level, participants requested less rushed clinical visits, more patient navigators who are living with HIV, more clinical appointment availability, more family-oriented care (i.e., allowing partners or other family receive concurrent care), more interactivity and greater privacy during appointment waiting periods, additional support groups (including flexible, remote options), advanced notice before care discontinuity, improved transportation support, co-located services and updated facilities. Participants expressed interest in having opportunities to provide similar feedback to health care providers. When asked, “what could providers or other support services do to make things better?” A participant responded, “Having more focus groups…. Not necessarily for the researchers. Maybe some doctors should come and sit in on a few of ‘em.”

Many patients took action in circumstances in which they perceived poor quality care. In some cases, participants proactively addressed their expectations for health care quality with providers at initial meeting, and for other participants, as quality issues arose. One focus group participants shared a preemptive strategy, “When I sit down and I talk to my provider, I’m gonna let you know exactly who I am, and if you feel you can’t deal with me, the individual that I am, then you step aside and get somebody who have a stronger back than you. When I say that I need something done, I need it done, and you’re not gonna say we’re gonna put this off when it come down to my health…. I’m serious about my life.”

Several participants advocated for themselves by requesting a new doctor or switching health care facilities when they were uncomfortable with or dissatisfied with aspects of care – particularly whether their preferences are being listened to or met, and how they were being treated. Accordingly, a participant relayed: “For me, one of the top 10 ways to die quick is to listen to your doctor unquestionably…. I just wouldn’t. It’d be like, I need a new doctor. That’s it, that’s all. I’m very wary of new people, especially those with the attitude of, I’m the physician and you’re the patient.” Alternatively, a few participants engaged in more formal action by reporting incidents to health care facility administration or to external parties.

Some participants described taking an advocacy role to empower other WLWH to confront or disengage from use of poorer than desired quality care. As a participant suggested, “… it ain’t nothin’ for us to be like, 'Okay, this will not happen again.' Basically, we won’t come back, but then it’s up to us to find another facility to go to or whatever. That’s the big fear they should have is that, when they have that bad experience, are they going to seek help again, or are they going to quit? They need to know that there’s somewhere they can go that they can talk to someone and say, ‘Hey, don’t let that discourage you. I got you.’ That’s what I do. I advocate for people...”

Other participants shared this sentiment and encouraged fellow focus group participants to use their voices in order to hold providers accountable around care quality. As a participant advised, "Maybe if you let them know, ‘cause maybe they don’t know how they’re letting us feel. I think that we’re in this field for so long that I think we can voice our opinion or even our dislike or just, “Listen. You are makin’ me feel uncomfortable…”.

## Discussion

Women living with HIV are a key population for which tailored HIV and other health care services are recommended in order to most effectively address the U.S. HIV epidemic. Successful epidemic response efforts will need to confront the distinct and intersecting social and systemic barriers to HIV-related and other health care services, considering the unique social positions that WLWH hold [7, 10]. The findings of this study indicate that women’s degree of care satisfaction was driven by whether care was knowledge-based and resulted in desired care outcomes (effectiveness); the coordination, continuity and necessity of care (efficiency); by disparities in care (equity); by how well care was delivered with compassion, nonjudgment, accommodation, and through shared decision-making processes (patient-centeredness); with attention to avoiding side effects and over medicalization (safety); and with limited wait time (timeliness). Results highlight that the six quality of care aims devised by the National Academy of Medicine in 2004 continue to be a potentially relevant framework for assessing the degree to which patient desires are met [34], including among women living with HIV, though it has seldom been employed to comprehensively assess patients’ perspectives in this population. Our study also contributes patient perspectives on how WLWH would prefer to see gaps in the health care to be improved.

In line with preferences for care that is knowledge-based and results-driven, as expressed by WLWH in our study, a Cochrane systematic review suggests that care from providers with training or expertise in HIV care results in better health outcomes as compared to care from providers who have less training or lower caseloads in HIV care [35]. Recent studies have identified gaps in provider knowledge of HIV-related services, and in the actual provision of those HIV-related services to women [36, 37]. Thus, provider knowledge has been a focal point of HIV prevention and treatment interventions, with demonstrated success in improving outcomes along the HIV prevention and treatment care continuum [37, 38]. Beyond HIV-specific medical education, training and development in structural competency may be particularly salient for medical professionals who serve Black and Latina WLWH because such trainings show promise in preparing providers to address aspects of the distinct and overlapping social and institutional barriers to health experienced by WLWH [39, 40].

HIV quality improvement initiatives have historically focused on the achievement of some of the outcomes that were discussed criterion for care satisfaction among the WLWH in the present study, such as improved clinic flow, mental health screening, and achieving ART adherence [41]. However, our study also highlighted health care treatment outcomes that patients reported were highly important to them but often not necessarily resolved for WLWH by clinic visits, such as pain reduction, that are not measured as part of many published HIV quality improvement studies. This divergence may reflect reliance upon quantitative performance measures in quality improvement efforts, potentially for efficiency, practicality, and to facilitate the monitoring of trends over time. Yet, the facets that are deemed to encompass quality of care, as important to care quality, as worthy of introspection, as needing to change, and by what standards, are all products of the perspectives that are represented is shaping quality frameworks, standards, and measurement. One of the benefits of the National Academy of Medicine quality of care framework that we leveraged to frame our emergent study findings is that the framework is broad enough for assessment via rich qualitative data, integrating the patient perspectives of Black and Latina WLWH. Notably, this and other commonly used frameworks for care quality are limited by lack of emphasis on health systems elements of quality of care including the financing, leadership and governance of health facilities [15].

The desire for integrated and coordinated care expressed by the WLWH in our study echoes existing literature, which proposes women-centered care models as a potential path toward minimizing gaps in the quality of care provided to WLWH [22–24]. The 30 for 30 Campaign - an effort by leading HIV service organizations dedicated to WLWH and women affected by HIV – defines women-centered care as “care that treats women holistically, [views] and [responds] to [women’s] needs with conscious attention to their real life circumstances” [42]. The campaign suggests that women’s use and the effectiveness of HIV related services is predicated in part upon the integration of HIV care services with other health and social support services, and the availability of wraparound and facilitative services (i.e., psychosocial services, transportation assistance, peer support, etc.). Furthermore, such services are more resource-intensive than most existing U.S. care delivery models, but potentially more cost effective if they are more successful in terms of women’s care experiences and outcomes.

Taken together, quality improvement work in all domains may be leveraged to achieve greater equity in health care experiences of WLWH, and as a result, in the health care engagement and health outcomes of WLWH. Considering lack of equity in these outcomes [43], attention to intersectionality is needed in the implementation of solutions. More specifically, an intersectional approach to addressing inequity requires centering the voices of marginalized persons and addressing multiple, converging systems of oppression [44], which warrants participatory praxis [25]. The structural competency framework also provides a lens within which health care providers and institutions can not only better recognize intersecting systemic inequities, but also address them in and outside of clinical practice [8]. Specified approaches to applying structural competency include greater education around the structural factors that shape clinical encounters (i.e., “group-differentiated access to goods, services, and resources”), greater discernment and articulation around the clinical presentations and outcomes in terms of the structural factors that produce them (e.g., lack of viral suppression rooted in racial discrimination [10]), and potential participation in and support for structural intervention (e.g., partnership with and resource allocation to community-based organizations leading anti-racist and WLWH -affirming work). Thus, participatory and structurally competent quality improvement initiatives may have greater potential for sustainability and impact in the direction of health equity.

As it pertains to participants’ desire for more compassionate and nonjudgmental care, a recent qualitative systematic review of 41 U.S. based studies found that the desire for respectful, empathetic care, that is de-stigmatizing and welcoming of diverse clients, were quality indicators that were important to engagement in primary care for persons living with HIV across studies [45]. Attempts to intervene upon provider stigma, and related attitudes and norms, reveal that they are potentially modifiable among providers in health professions [32]. Regarding timeliness, other U.S. based studies have also documented similar concerns about the length of wait times both during clinic visits and between available appointments [46]. Efforts to provide HIV care remotely through telehealth in Veterans Health Administration facilities have been preferred by people living with HIV relative to in person care, in part because of shorter wait times, but also for the convenience of not having to travel or take as much time away from work or other responsibilities. Patients also reported appreciating that telehealth care better protected their privacy [47].

Limitations of this study include that this study examined quality of care among clients who were enrolled in a research cohort study. Perspectives on quality of care may differ between women who participated in our study and those who are not engaged in a system of research and care such as the WIHS. Nevertheless, women in our study shared prior personal experience with lack of care engagement, and at times, shared that of other acquaintances – providing a diversity of voiced experiences. Finally, while the focus groups and interviews conducted for this study were guided by an English and Spanish version of the same instrument, the dynamics are understandably different within focus groups as compared to one-on-one interviews. Additionally, both focus groups and interviews can yield social desirability and biases influenced by multiple factors, including level of comfort with sharing perspectives in social settings. That said, the study moderators and interviewers employed strategies during focus groups to promote equitable engagement such as asking focus group participants to share speaking time with others and calling on participants who had less opportunity to speak.

## Conclusion

Quality of care represents an important and changeable lever affecting the ability and desire for WLWH to engage in care [9, 10]. The communities most proximally affected by HIV should be key stakeholders in HIV-related quality assurance [48]. Our study contributes to the existing scientific literature through a qualitative investigation of perceptions of healthcare among Black and Latina women living with HIV in six U.S. cities. This study expands the scope of prior U.S.-based HIV-related care quality research, which has largely focused on quantitative clinical quality indicators. This study also adds a comprehensive assessment across all of the National Academy of Medicine care quality domains, from the perspectives and lived experiences of minority women living with HIV. Findings highlight the importance of incorporating WLWH in patient centered, participatory quality improvement initiatives, consistent with emerging women-centered healthcare models which meaningfully involve WLWH throughout the health care design and delivery process in the forms of outreach and consult through collaboration and shared leadership. Such participatory models may be most responsive in addressing the intersecting social and systemic inequities facing Black and Latina WLWH with structural competency.

## Data Availability

The data generated during the current study are not publicly available because study participants who participated did not consent to the data being made available to other researchers. However, we have provided the focus group and interview guides used to collect data for this study (see Additional files 1 and 2). Furthermore, additional qualitative data from our dataset are being prepared for publication elsewhere.

## Abbreviations

ART: Antiretroviral therapy
COPD: Chronic obstructive pulmonary disease
HIV: Human immunodeficiency virus
TB: Tuberculosis
WIHS: Women’s Interagency HIV Study
WLWH: Women living with HIV
UNC: University of North Carolina at Chapel Hill
US: United States
